# Hyperthermia reduces migration of osteosarcoma by suppression of autocrine motility factor

**DOI:** 10.3892/or.2012.2066

**Published:** 2012-10-01

**Authors:** KOSEI NAKAJIMA, TAKASHI YANAGAWA, HIDEOMI WATANABE, KENJI TAKAGISHI

**Affiliations:** 1 Department of Orthopedic Surgery, Graduate School of Medicine, Gunma University, Maebashi, Gunma, Japan; 2 Department of Physical Therapy, Graduate School of Health Science, Gunma University, Maebashi, Gunma, Japan

**Keywords:** autocrine motility factor, heat shock protein, hyperthermia, metastasis, motility, osteosarcoma

## Abstract

Autocrine motility factor (AMF) plays an important role in the development of metastasis by regulating tumor cell motility. The expression of AMF is associated with metastasis in malignant musculoskeletal tumors including osteosarcoma. Recent studies indicated that hyperthermia contributes to the improvement of the prognosis of patients with soft tissue sarcomas; however, few reports have evaluated the impact of hyperthermia on tumor cell motility, which is an important factor of metastasis. The purpose of this study was to evaluate the effect of hyperthermia with or without heat shock protein (HSP) inhibitors on the motility and AMF expression in an osteosarcoma cell line. Hyperthermia was carried out at 41°C for 24 h. According to microarray results, HSP90, HSP70 and HSP27 expression was upregulated in osteosarcoma cells under hyperthermia. The intracellular, secreted AMF, mRNA of AMF and cell motility were evaluated by western blotting, ELISA, RT-PCR, wound healing and phagokinetic track assays, respectively. The protein secretion and mRNA levels of AMF and tumor cell motility were significantly decreased by hyperthermia. Of note, the downregulated AMF expression and motility were recovered by the addition of an HSP27 inhibitor. By contrast, the HSP90 and HSP70/72/105 inhibitors had no effect on AMF expression and motility downregulated by hyperthermia. In conclusion, hyperthermia reduced AMF expression and tumor cell motility via HSP27 and may therefore be applied as osteosarcoma treatment.

## Introduction

Osteosarcoma is the most common primary malignant bone tumor; it occurs during adolescence and is ranked eighth in general incidence among childhood cancers. The overall 5-year survival rate for osteosarcoma ranges from 50 to 80% (1–5). Despite adequate surgical removal and chemotherapy at an early stage of osteosarcoma, some patients experience distant metastasis to the lung, bone or other organs; therefore, prevention of the invasion and metastasis of osteosarcoma is crucial in improving the prognosis of patients. To combat the aggressive potential of osteosarcoma, various factors associated with invasion and metastasis have been identified. Among them, autocrine motility factor (AMF) plays an important role in metastasis. AMF is secreted by tumor cells and stimulates the potential of proliferation (6), migration (7,8), angiogenesis (9,10) and resistance to apoptosis (11,12). In previous studies, molecular cloning and sequencing identified phosphoglucose isomerase (PGI) as AMF (13). PGI is an essential cytosolic enzyme of sugar metabolism and plays a key role in both glycolysis and the gluconeogenesis pathway, catalyzing the interconversion of glucose 6-phosphate and fructose 6-phosphate in both normal and tumor cells (14). PGI is secreted extracellularly from various tumors, not from normal cells, and behaves as AMF (15–17). Elevated serum AMF levels in malignant tumors, including the gastrointestinal tract (18), colorectum (19), breast (20), and lung cancer (21), are associated with cancer progression and metastasis. In addition, recent studies indicated that upregulated AMF expression is involved in the metastasis of osteosarcoma (22). Moreover, silencing AMF causes mesenchymal-to-epithelial transition and completely prevents pulmonary metastasis of osteosarcoma (23). Thus, suppressing AMF appears to be an appropriate treatment to control tumor invasion and metastasis; however, inhibition of AMF expression throughout the body may have a risk. AMF works as PGI, which is a critical molecule for glucose metabolism in all kinds of cells and, therefore, suppressing AMF in the whole body may inhibit a patient’s ability to metabolize glucose. This is one of the reasons why clinical trials to suppress AMF have not been performed.

Hyperthermia is an effective local adjuvant therapy for carcinomas and sarcomas. A randomized phase III trial showed that regional hyperthermia combined with neo-adjuvant chemotherapy for soft tissue sarcomas had better local progression-free survival than chemotherapy alone (24). Several reports have suggested that hyperthermia for osteosarcoma achieved an effective response, including the induction of apoptosis (25) and inhibition of tumor proliferation (26,27) and DNA synthesis (27)*in vitro*. Regional hyperthermia using an alternating magnetic field reduced the pulmonary metastasis of osteosarcoma in an *in vivo* study (28). In the present study, we examined the involvement of AMF and heat shock genes including heat shock protein (HSP) and tumor cell motility in osteosarcoma cells under normal and hyperthermic conditions.

## Materials and methods

### Antibodies and reagents

Anti-AMF/PGI mouse monoclonal antibody was purchased from ProMab Biotechnologies Inc. (Richmond, CA, USA) and anti-β-actin mouse monoclonal antibody was purchased from Sigma-Aldrich Inc. (St. Louis, MO, USA). 17-AAG, a heat shock protein (HSP)90 inhibitor, KNK437, an HSP70/72/105 inhibitor, and KRIBB-III, an HSP27 inhibitor were purchased from Selleck Chemicals Inc. (Houston, TX, USA), Merck Inc. (Darmstadt, Germany) and Sigma-Aldrich Inc., respectively. The horseradish peroxidase (HRP)-conjugated goat anti-mouse antibody was purchased from Zymed Inc. (South San Francisco, CA, USA). The enzyme-linked immunosorbent assay kit for human glucose 6 phosphate isomerase was purchased from Uscn Life Science Inc. (Wuhan, China).

### Cell culture

The human osteosarcoma cell line HuO9 was kindly provided by Dr T. Hotta (Niigata University, Niigata, Japan) and grown in RPMI-1640 supplemented with 10% heat-inactivated fetal bovine serum (FBS). The cells were maintained at 37°C in a humidified atmosphere of 5% CO_2_ and 95% air.

### Treatment with hyperthermia and HSP inhibitors

Culture with hyperthermia was carried out at 41°C for 24 h in a 5% CO_2_ incubator. Prior to hyperthermia exposure, cells were washed with phosphate-buffered saline (PBS), and fresh medium was added. The concentrations of HSP inhibitors were less than the cytotoxic level shown in previous reports, with 10 nM for 17-AAG (29) and KRIBB-III (30) and 10 μM for KNK437 (31).

### DNA microarray analysis

HuO9 cells were separated into two conditions, 41 and 37°C. The isolated total-RNA of the cells in each condition was used for synthesis of cDNA, which was labeled with biotin and hybridized with the GeneChip Array, Human Genome U133 Plus 2.0 Array (Affymetrix Inc., Santa Clara, CA, USA). The array was scanned with a GeneChip 3000 scanner. The signal intensities from hybridized cDNA were quantified. The final processed data were obtained by the global normalization method using GCOS software.

### RT-PCR analysis

Total-RNA was isolated from hyperthermia-treated HuO9 cells with or without HSP inhibitors for 24 h using Isogen (Wako Pure Chemical Industries, Osaka, Japan). The cDNA was generated using a SuperScript III First-strand Synthesis SuperMix (Invitrogen Inc., Carlsbad, CA, USA) as recommended in the manufacturer’s protocol. The products of reverse transcription reactions were used for PCR. β-actin was used as an internal control. The number of amplification cycles for PGI/AMF, β-actin genes, was 25, respectively, which was selected to allow linear amplification of the cDNA under study. The primer sequences and their respective PCR fragment lengths were: PGI/AMF, 5′-AATGCAGAGACGGCGAAGAAG-3′ (forward) and 5′-ACGAGAAGAGAAAGGGGAGTC-3′ (reverse) (1066 bp); β-actin, 5′-TGACGCGGTCACCCACACTGTGCCCAT-3′ (forward) and 5′-CTAGAAGCATTTGCGGTGGGAGGG-3′ (reverse) (610 bp). PCR products were electrophoresed on 1% agarose gels, stained with ethidium bromide and photographed.

### Sampling intracellular AMF from cell cultures

HuO9 cells cultured on 10-cm dishes were treated by hyperthermia with or without HSP inhibitors for 24 h and then transferred to 37°C for 24 h in a 5% CO_2_ incubator. Intracellular proteins were collected by scraping and lysed in radioimmune precipitation assay buffer (20 mM Tris-HCl, pH 7.4, 150 mM NaCl, 10 mM EDTA, 1% of NP-40, Triton X-100, sodium deoxycholate) containing 1 mM phenylmethylsulfonyl fluoride. After cell lysates were centrifuged, the supernatants were subjected to SDS-PAGE to investigate the expression of intracellular AMF/PGI and β-actin. The protein concentration of each sample was determined using Bio-Rad protein assay reagent (Bio-Rad Laboratories Inc., Hercules, CA, USA).

### Western blot analysis

All protein samples were separated on 10% SDS-PAGE gels and transferred to a polyvinylidene difluoride membrane (Millipore Inc., Billerica, MA, USA). Western blotting was carried out by the SNAP-id protein detection system (Millipore Inc.) according to the manufacturer’s instructions. The membrane was blocked with Bløk, a noise-cancelling reagent (Millipore Inc.), for 30 sec at room temperature. The blocked membrane was incubated with diluted primary antibodies (AMF/PGI 1:1,000, β-actin 1:1,000) for 10 min. Following extensive washing, anti-mouse HRP-conjugated secondary antibody (1:1,000) was added and incubated for 10 min. Proteins were visualized using a chemiluminescence (ECL) system.

### ELISA

To examine the concentration of secreted AMF, an enzyme-linked immunosorbent assay kit for human glucose 6 phosphate isomerase (Uscn Life Science Inc.) was used according to the manufacturer’s instructions. The secreted protein was isolated from RPMI with 10% FBS. HuO9 cells were expanded on 10-cm dishes as a confluent monolayer. After the cells had been exposed to hyperthermia with or without HSP inhibitors for 24 h, the medium was replaced with 5 ml RPMI with 10% FBS. Supernatants were collected for ELISA after the cells had been incubated at 37°C for 24 h.

### Wound healing assay

Horizontal motility was measured by the wound-healing assay. The surface of the cultured HuO9 cell monolayer in each prepared well was wounded by a pipette tip and the medium was replaced. After 24 and 48 h of incubation, the wounded area was photographed and the wound area filled was calculated using the formula: % wound area filled = (average wound width before incubation - average wound width after incubation / average wound width before incubation) × 100.

### Phagokinetic track assay

Random cell motility was measured by the phagokinetic track assay as previously described. Uniform carpets of gold particles were prepared on coverslips coated with 1.0% bovine serum albumin (BSA) by fixing with 100% ethanol and warm air drying. The treated coverslips were then embedded with colloidal gold particles and placed in 35-mm tissue culture dishes. Then, 3,000 cells in suspension culture were added to the plates. After 24 h, the phagokinetic tracks were visualized using dark field illumination with a Nikon inverted microscope. The area cleared of gold particles by ≥30 cells was measured using NIH Image J.

### Statistical analysis

Statistical analysis was performed using SPSS 17.0 software (SPSS Inc., Chicago, IL, USA). Wound healing and phagokinetic track motility assays were analyzed using analysis of variance (ANOVA) and the significance of individual differences was evaluated using the Tukey HSD if ANOVA was significant.

## Results

### Upregulated heat shock proteins in osteosarcoma cells under hyperthermia

HSP has several subtypes, HSP110/105, HSP90, HSP72/70, HSP60, HSP40, HSP27, HSP22, and HSP10. We confirmed the molecular responses of HuO9 cells under hyperthermia using a microarray. More than 2-fold upregulated genes among HSPs compared to the control were HSP105, HSP70, HSP27 and HSP22 (Table I). Since there are no reports showing that HSP22 is involved in cell migration, cancer metastasis, invasion, or proliferation, KNK437, an HSP70/72/105 inhibitor, and KRIBB-III, an HSP27 inhibitor, were selected in this study. 17AAG, an HSP90 inhibitor, was also selected as this subtype has been well examined as a molecular target drug for cancer therapy (32,33), although the increase of HSP90 gene expression was slight (1.82-fold).

### Hyperthermia reduces mRNA level and secretion of AMF

**Figure 1:**
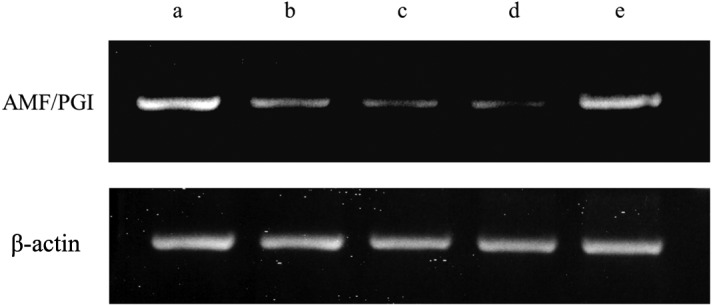
AMF mRNA expression of the HuO9 osteosarcoma cell line under hyperthermia with or without HSP inhibitors. Heat shock induced AMF mRNA suppression, which was recovered by the HSP27 inhibitor, KRIBB-III. Representative results of 3 different experiments are shown. a, control; b, hyperthermia; c, hyperthermia + 17-AAG; d, hyperthermia + KNK437; e, hyperthermia + KRIBB-III.

**Figure 2:**
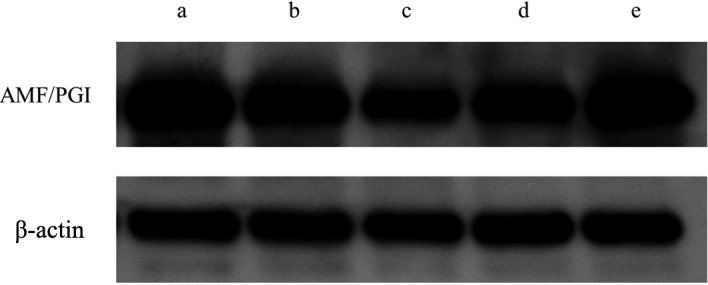
Intracellular AMF expression of the HuO9 osteosarcoma cell line under hyperthermia with or without HSP inhibitors. Similar to mRNA, intracellular AMF was reduced by hyperthermia and recovered by the HSP27 inhibitor. Representative results of 3 different experiments are shown. a, control; b, hyperthermia; c, hyperthermia + 17-AAG; d, hyperthermia + KNK437; e, hyperthermia + KRIBB-III.

**Figure 3:**
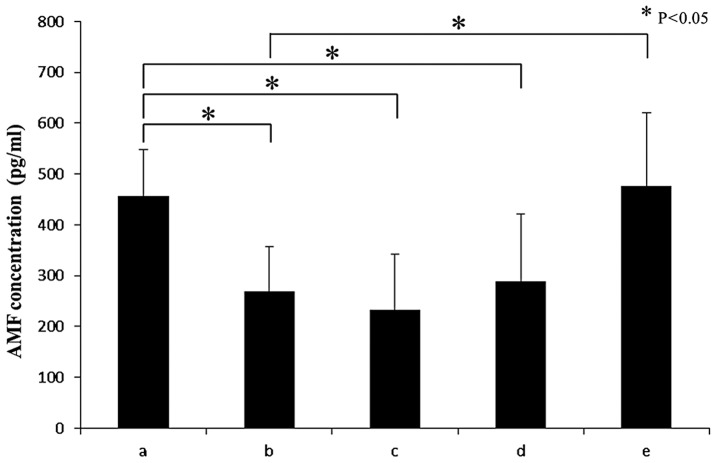
Secreted AMF levels measured with ELISA under hyperthermia with or without HSP inhibitors. AMF secretion was inhibited by hyperthermia and recovered to the level of the control by the addition of HSP27. Data are presented as the mean ± SD of 6 different experiments. a, control; b, hyperthermia; c, hyperthermia + 17-AAG; d, hyperthermia + KNK437; e, hyperthermia + KRIBB-III.

To analyze the effect of hyperthermia on AMF expression, the HuO9 osteosarcoma cell line was exposed to hyperthermia at 41°C and to normal conditions at 37°C for 24 h. The level of mRNA, intracellular protein and secreted protein was determined by RT-PCR, western blotting and ELISA, respectively. The HuO9 osteosarcoma cell line showed a decrease in the mRNA level of AMF under hyperthermic conditions (Fig. 1). Western blotting revealed slight downregulation of intracellular AMF compared with the control sample (Fig. 2). Secreted AMF measured with ELISA was significantly downregulated by hyperthermia compared with the control [hyperthermia; mean 269.0±87.4 (SD) vs control; mean 456.7±90.5 p=0.047] (Fig. 3). Previous reports showed that the amount of secreted AMF was mainly dependent on the mRNA level, not on the intracellular protein level (15–17); therefore, we concluded that hyperthermia reduced the expression of mRNA of AMF and subsequently suppressed the secretion of AMF.

### Hyperthermia reduces the motility of osteosarcoma

**Figure 4:**
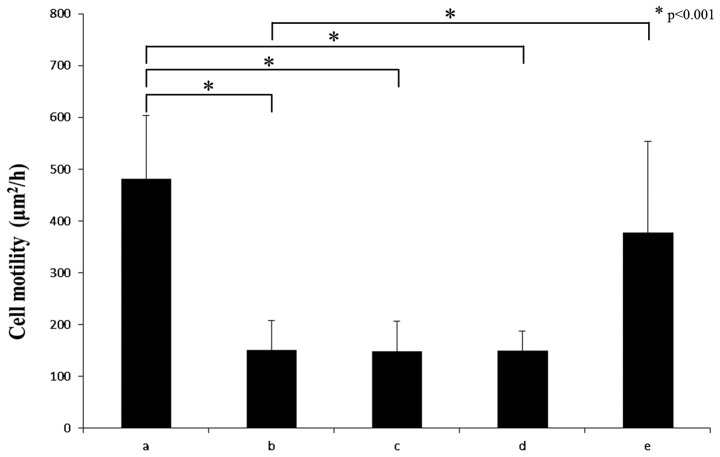
The results of a phagokinetic track assay for HuO9 osteosarcoma cells incubated under hyperthermia with or without HSP inhibitors. The motility paralleled the AMF expression presented in Figs. 1–3. Asterisk (^*^) indicates a significant difference from the control group (p<0.001). a, control; b, hyperthermia; c, hyperthermia + 17-AAG; d, hyperthermia + KNK437; e, hyperthermia + KRIBB-III.

**Figure 5:**
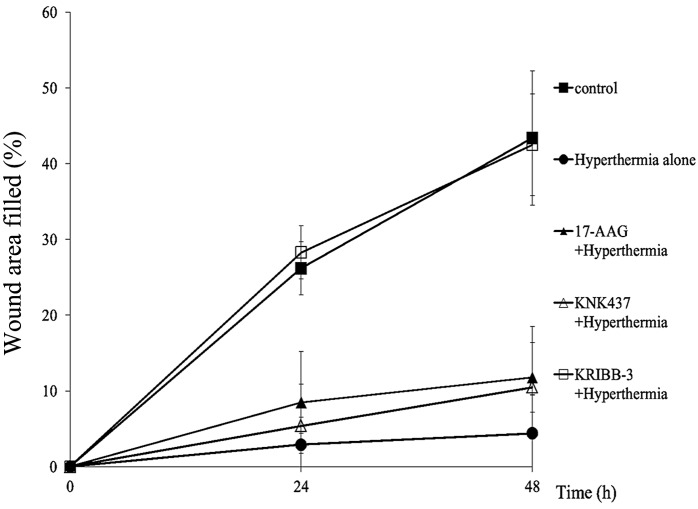
Results of the wound healing assay. Graph shows the effect of hyperthermia with or without HSP inhibitors on the cell motility of the HuO9 osteosarcoma cell line. Results were quantified by NIH Image J. Data are presented as the mean ± SD of 3 different experiments.

The motility of osteosarcoma cells was significantly decreased under hyperthermia compared to the control sample at a normal temperature. The phagokinetic track assay showed that random motility of HuO9 cells was significantly suppressed by hyperthermia (p<0.001) (Fig. 4). Furthermore, the wound-healing assay revealed the significant suppression of horizontal motility under hyperthermia. The difference was apparent at 24 h (hyperthermia; mean 2.9±3.6 vs control; mean 26.2±3.5 p=0.004) and at 48 h (hyperthermia; mean 4.4±5.1 vs control; mean 43.4±8.9 p=0.001) after hyperthermia (Fig. 5).

### Effect of HSP inhibitors on AMF expression and motility under hyperthermic and normal conditions

**Figure 6:**
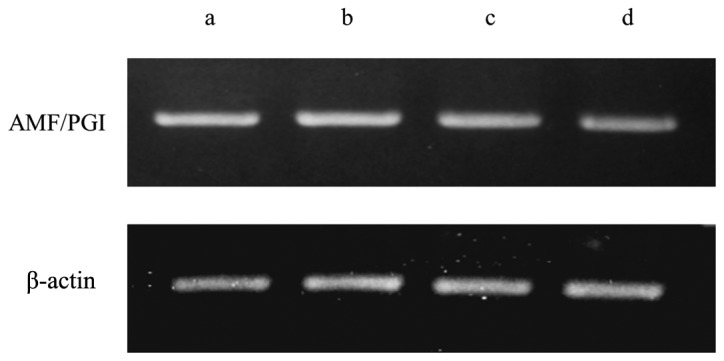
AMF mRNA expression of the HuO9 osteosarcoma cell line under normal conditions with or without HSP inhibitors. The expression of AMF was not affected by HSP inhibitors under normal conditions. Representative results of 3 different experiments are shown. a, control; b, hyperthermia + 17-AAG; c, hyperthermia + KNK437; d, hyperthermia + KRIBB-III.

To explore which HSP pathways play an important role in the regulation of AMF under hyperthermia, we treated HuO9 cells with various HSP inhibitors during hyperthermic exposure. The AMF mRNA level downregulated by hyperthermia was recovered to almost the same level at 37°C by the addition of an HSP27 inhibitor, KRIBB-III (Fig. 1). Western blotting and ELISA also showed the recovery of intracellular and secreted AMF by the addition of KRIBB-III under hyperthermic conditions, as expected (p=0.024) (Figs. 2 and 3). These results suggest that the downregulation of AMF expression under hyperthermia is associated with HSP27. As shown in Figs. 4 and 5, the recovery of cell motility was observed under hyperthermic conditions with KRIBB-III by the phagokinetic track (p<0.001) and wound-healing assay, respectively. Meanwhile, an HSP90 inhibitor, 17-AAG, and an HSP70/72/105 inhibitor, KNK437, had no statistically significant effect on AMF expression and motility compared with hyperthermia alone. No HSP inhibitors affected AMF expression under normal conditions (Fig. 6).

## Discussion

In the present study, we discovered that hyperthermia reduced the mRNA level of AMF, secreted AMF and the motility of osteosarcoma cells. The difference in the amount of secreted AMF between the hyperthermia-treated sample and control was approximately 180 pg/ml, which appeared sufficient to suppress cell motility since a previous study indicated that addition of AMF 100 pg/ml enhanced motility by 1.5-fold (7,13). A few reports have described the mechanisms of how hyperthermia affects tumor cell migration, although there have been many reports on thermal therapy preventing tumor cell proliferation. Sato *et al* reported that hyperthermia suppressed the invasion of fibrosarcoma by inhibiting the production of membrane type-1 matrix metalloproteinase (MMP) and proMMP-2 activity (34). In the present study, we found that a new mechanism was involved in the suppression of osteosarcoma cell motility under hyperthermia via AMF downregulation.

To date, no clinically available agents inhibiting AMF have been reported, although some articles have reported that silencing AMF by RNA interference (35,36) or hammerhead ribozyme (23) could inhibit metastasis and the invasion of malignant tumors. Since AMF works as PGI, which plays an important role in glucose metabolism not only in tumors but also in normal cells (15,37,38), complete block of AMF/PGI throughout the body may be harmful or even lethal for normal cells, compelling us to identify procedures that regulate AMF expression locally. Hyperthermia is well known as a clinically available modality for cancer therapy. Regional hyperthermia combined with neo-adjuvant chemotherapy for soft-tissue sarcomas showed better local progression-free survival than chemotherapy alone in a randomized study (24). Hyperthermic isolated limb perfusion with pre-operative chemotherapy for osteosarcoma patients achieved a good response compared to chemotherapy without hyperthermia (39). We expect that regional hyperthermia of osteosarcomas can control AMF secretion from tumor cells without affecting the glucose metabolism throughout the body and prevent tumor invasion and metastasis.

Little is known about the regulatory mechanism of AMF expression as promoter region analysis of AMF remains insufficient, except for one report showing that a minisatellite in intron 9 of human PGI genes stimulated transcription from PGI promoter (40). Hypoxia induces AMF expression via HIF-1α (41); however, there have been no reports on the proteins or conditions that downregulate AMF expression. In our study, reduced AMF expression by hyperthermia was recovered by the addition of the HSP27 inhibitor, which indicates that HSP27 inhibits AMF expression and tumor cell motility under hyperthermia.

HSPs are induced not only by heat shock but also by other pathological conditions and work as molecular chaperones in maintaining cellular homeostasis and contributing to cell survival (42). Mammalian HSPs have been classified into six groups and HSP27 belongs to small HSPs. Largely oligomerized HSP27 works as a chaperone preventing aggregation while a small oligomer of this molecule stabilizes actin filaments (43). In immunohistochemical studies, overexpression of HSP27 was associated with poor prognosis (44) and distant metastasis (45) in osteosarcoma patients and was found at a higher rate in high-grade than low-grade osteosarcomas (45). HSP27 expression was associated with favorable prognosis only in malignant fibrous histiocytoma among many types of cancer (46). Shin *et al* reported that the HSP27 inhibitor inhibited cancer proliferation and migration by blocking HSP27 phosphorylation under normal conditions (30). There have been several reports on the use of the HSP27 inhibitor under normal conditions, but there are no reports on its use under hyperthermia. We speculated that the discrepancy in the effects of HSP27 on tumor cell migration between our results and previous reports is likely due to a difference in HSP27 function between normal and hyperthermic conditions. Hyperthermia treatment presents a theoretical dilemma; heat shock stress induces various heat shock proteins, the expression of which is related to poor prognosis and the inhibitors of which are clinically used for cancer therapy (43), although thermal treatments have achieved favorable treatment results. Our hypothesis that HSP27 functions depend on temperature (normal or hyperthermia) may resolve the dilemma of hyperthermia treatments. In addition, hyperthermia is expected to be more effective for HSP27-expressing osteosarcoma as HSP27 has a propensity to suppress tumor migration under hyperthermic conditions.

In conclusion, hyperthermia suppressed the expression of AMF and the motility of the HuO9 osteosarcoma cell line via HSP27. Our results suggest that hyperthermia is effective in preventing the invasion and metastasis of osteosarcoma by reducing AMF, and HSP27 regulates AMF expression under hyperthermia. Therefore, hyperthermia may be a clinical therapeutic modality for osteosarcoma in, for example, adjuvant and palliative therapies.

## Figures and Tables

**Table I:** HSP genes >2-fold upregulated by hyperthermia compared with the control sample.

Gene name	Gene symbol	GenBank accession no.	Fold change
Heat shock 105-kDa/110kDa protein 1	HSPH1	NM_006644	2.77
Heat shock 70-kDa protein 1A	HSPA1A	NM_005345	4.45
Heat shock 70-kDa protein 1B	HSPA1B	NM_005346	4.4
Heat shock 70-kDa protein 6 (HSP70B′)	HSPA6	NM_002155	6.67
Heat shock 27-kDa protein 1	HSPB1	NM_001540	2.84
Heat shock 22-kDa protein 8	HSPB8	AF133207	2.41

## Abbreviations

AMF: autocrine motility factor
PGI: phosphoglucose isomerase
HSP: heat shock protein
MMP: matrix metalloproteinase
